# Birth weight centiles by gestational age for twins born in south India

**DOI:** 10.1186/s12884-016-0850-y

**Published:** 2016-03-24

**Authors:** Prasanna Premkumar, Belavendra Antonisamy, Jiji Mathews, Santhosh Benjamin, Annie Regi, Ruby Jose, Anil Kuruvilla, Mathews Mathai

**Affiliations:** Departments of Biostatistics, Christian Medical College, Vellore, 632 002 India; Obstetrics and Gynaecology, Christian Medical College, Vellore, 632 002 India; Neonatology, Christian Medical College, Vellore, 632 002 India; Making Pregnancy Safer Department, World Health Organization, Geneva, Switzerland

**Keywords:** Twins, Birth weight, Gestational age, Centiles, India, GAMLSS

## Abstract

**Background:**

Birth weight centile curves are commonly used as a screening tool and to assess the position of a newborn on a given reference distribution. Birth weight of twins are known to be less than those of comparable singletons and twin-specific birth weight centile curves are recommended for use. In this study, we aim to construct gestational age specific birth weight centile curves for twins born in south India.

**Methods:**

The study was conducted at the Christian Medical College, Vellore, south India. The birth records of all consecutive pregnancies resulting in twin births between 1991 and 2005 were reviewed. Only live twin births between 24 and 42 weeks of gestation were included. Birth weight centiles for gestational age were obtained using the methodology of generalized additive models for location, scale and shape (GAMLSS). Centiles curves were obtained separately for monochorionic and dichorionic twins.

**Results:**

Of 1530 twin pregnancies delivered during the study period (1991–2005), 1304 were included in the analysis. The median gestational age at birth was 36 weeks (1st quartile 34, 3rd quartile 38 weeks). Smoothed percentile curves for birth weight by gestational age increased progressively till 38 weeks and levels off thereafter. Compared with dichorionic twins, monochorionic twins had lower birth weight for gestational age from after 27 weeks.

**Conclusions:**

We provide centile values of birth weight at 24 to 42 completed weeks of gestation for twins born in south India. These charts could be used both in routine clinical assessments and epidemiological studies.

## Background

Rate of twin births is rising due to the increased use of assisted reproductive technologies in the recent years [1]. Birth weight of twins are considerably lower than singletons and associated with higher risk for adverse perinatal and infant outcomes [2, 3]. Birth weight centiles by gestational age is often used as a health indicator and to understand the natural extent of variation in birth weight. There have been many studies of twin birth weight centiles worldwide [4–7], but are of limited use in developing settings like India owing to the use of non-representative populations. In India, information on twins is quite limited, and most previous studies on centile curves focused only on singleton births [8–10]. Currently, the clinical practice is that centiles curves for singleton births are used as an estimate to evaluate twin births. However, recent studies suggest use of singleton centile curves on twins is not appropriate as twins experience different growth trajectories than singletons [11]. Moreover, several studies have recommended the development of twin specific centile curves to evaluate twin births [12–15].

Twin births are further complicated by placental chorionicity. Monochorionic twins present a two to three times higher risk for adverse outcomes than dichorionic twins [16], with birth weights of monochorionic twins lower than those of dichorionic twins over the gestational ages. Further, monochorionic placentation increases the risk of serious pregnancy complications (such as twin-to-twin transfusion syndrome), congenital anomalies, growth restriction, and perinatal death [17].

Past studies have indicated that placental chorionicity should be taken into consideration in assessment of twin births [18]. The lack of centile curves specific to twins could be a limiting factor in understanding the distribution of birth weight and further evaluation of twin births. Therefore, we carried out the present study to construct gestational age specific birth weight centile curves for twins born in South India, stratified by placental chorionicity (monochorionic and dichorionic placentation).

## Methods

### Setting and population

This study was based on labour room records and medical records maintained by the Department of Obstetrics and Gynaecology at the Christian Medical College, Vellore. This hospital serves as a maternity centre with almost 9000 deliveries annually (during the study period, 1991–2005). It provides obstetric care to local population of Vellore city and for surrounding towns and villages and also acts as a tertiary hospital. Besides women from Vellore district, women from neighbouring districts in Tamil Nadu and adjoining states of Andhra Pradesh and Karnataka also deliver in the institution. Most pregnancies are registered early during the first or second trimesters and followed up thereafter for antenatal care.

Ethical approval of the study protocol was obtained from the Institutional Review Board (IRB: 2000-no.4481) of the Christian Medical College, Vellore. However, because of the retrospective nature of the study and data were extracted from medical records/labour room registers with no individual identifications, and hence individual informed consent was not obtained.

### Study sample

The study sample included all twin pregnancies delivered at the centre between January 1, 1991 and December 31, 2005. Pregnancies in which at least one child died, or missing information on study variables were excluded from further analysis.

### Study variables

Birth weight was measured immediately after birth on a Braun weighing scale to the nearest 50 g. Gestational age was determined as the number of completed weeks of gestation from the last menstrual period (LMP) to the date of birth. This was best estimated using combinations of the last menstrual period (LMP), early clinical examination and early ultrasound scans. If there was a difference between gestational age estimated from LMP and ultrasound, the ultrasound estimate was used. Placental chorionicity was diagnosed by ultrasound and confirmed by gross examination of placenta after the birth.

### Statistical analysis

From an initial exploratory analysis, we found that the distribution of birth weight at extreme gestational ages was non-normal and the general pattern of relationship between birth weight and gestational age was not linear. Given these violations in the usual assumptions of regression analyses, we chose the generalized additive model for location scale and shape (GAMLSS) approach [19]. This approach is highly flexible as it relaxes the traditional distributional assumptions about normality to include even highly skewed and kurtotic distributions. It extends not only to model mean but all other parameters (standard deviation, skewness and kurtosis) of the distribution as linear, non linear or smoothing functions of explanatory variables (gestational age). In our analyses, we have used Box-Cox t (BCT) distribution for modeling birth weight as non-parametric cubic spline functions of gestational age. Model selection was based on generalized Akaike Information Criterion (GAIC) and the model with smallest value of the GAIC is selected. Worm plots were used for visual inspection of the fit of the smoothed curves and were further confirmed by superimposing the smoothed centiles on observed empirical centiles. Centile curves were obtained for the entire sample and were also constructed according to placental chorionicity. The GAMLSS package for R statistical software (version 2.13.1) was used for the analysis [20].

## Results

During the study period, a total of 1673 multiple pregnancies were delivered. Of which, the following were removed sequentially from further analysis (triplets = 39; fetal deaths of one or more foetuses = 141; missing data on chorionicity = 170; and missing data on birth weight and/or gestational age = 19). Thus complete data were available from 1304 twin gestations for analyses. The mean maternal age of mothers included in the sample was 25.2 years (SD = 4.3) and 46 % of mothers were primigravid. A total of 88 (6.7 %) mothers were conceived using some form of assisted reproductive technologies, while approximately (64) 5 % of mothers experienced gestational diabetes and (224) 17 % had preeclampsia. Eighty two percent of the women in our sample were Hindus, 11 % were Muslims and 7 % were Christians. About 8 % (106) of mothers were illiterates. There were 457 (35 %) monochorionic pregnancies.

The median gestational age at birth was 36 weeks (IQR 34–38 weeks). There was a 4.1 % increase in adjusted (for gestational age) mean birth weight from 2050 g in 1991 to 2135 in 2005. Dichorionic twins were heavier than monochorionic twins with an adjusted (for gestational age) mean of 2138 g compared with 2, 054 g respectively. The mean birth weight discordance was 13.1 % (SD = 10.3 %, median = 11.1 %). Considering a threshold of 18 % [18], birth weight discordance was identified in 360 out of 1304 pregnancies (27.6 %).

**Fig. 1:**
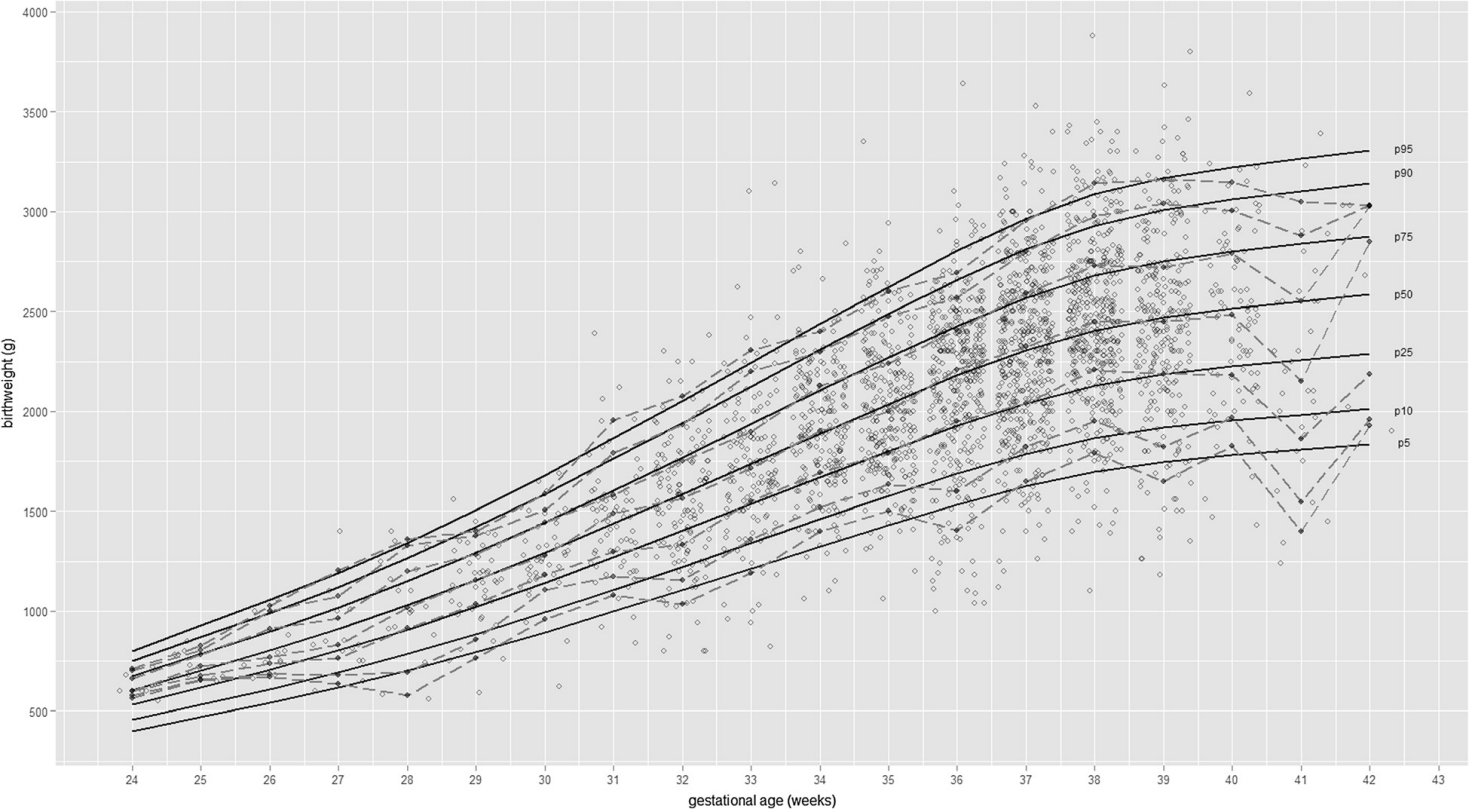
Smoothed centiles of birth weight for gestational age for the entire sample of twins (*solid lines*) and raw centiles (*dotted lines*). Points are jittered to improve readability

Examining the 50th centile, the weekly increase in birth weight flattens by 38 weeks of gestation and thereafter gain in the median birth weight was negligible (Fig. 1). To assess the validity of the fitted model, the expected percentage of observed birth weights below each centile was compared with observed percentage across gestational ages. About, 9 % fell below the 10th centile, 80.4 % between 10th and 90th centile and 9.9 % above the 90th centile. Further, the fit of the curves estimated from the statistical models were confirmed by overlaying the empirical centiles on top of the smoothed centiles (Fig. 1).

**Fig. 2:**
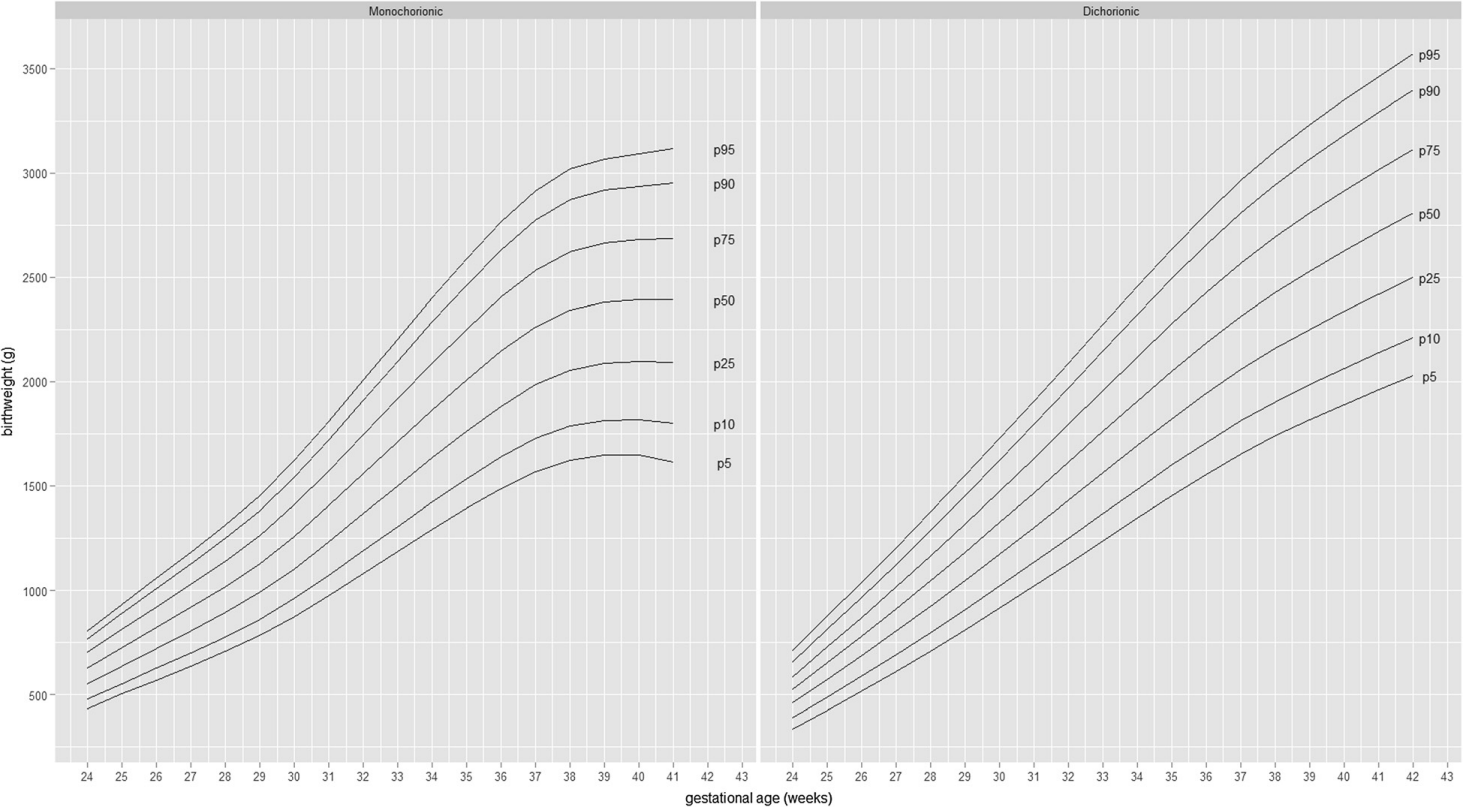
Smoothed centiles of birth weight for gestational age according to chorionic presentations

Figure 2 present birth weight centile curves for twins according to placental chorionicity. Monochorionic twins were consistently smaller than dichorionic twins after 27 weeks of gestation, with a fall-off across centiles of birthweight (Tables 1 and 2).

**Table 1:** Distribution of birthweight by gestational age (weeks) for twins according to chorionic placentation

	Monochorionic						Dichorionic					
GA	N	P10	P50	P90	Mean	SD	N	P10	P50	P90	Mean	SD
24	4	600	640	708	650	60.00	2	555	575	595	575	35.36
25	8	657	725	815	733.75	71.30	2	681	725	769	725	77.78
26	8	709	840	1018	860	147.45	4	698	740	775	737.5	45.00
27	2	756	780	804	780	42.43	12	660	840	1091	889.17	215.43
28	6	830	1120	1375	1108.33	264.08	12	610	1015	1205	987.5	230.26
29	26	785	1125	1300	1076.92	195.44	16	1060	1265	1425	1243.75	176.74
30	18	1052	1270	1503	1263.89	182.92	26	1130	1285	1550	1304.23	220.53
31	18	1075	1400	1645	1387.22	275.66	50	1189	1500	1823	1504.2	275.19
32	54	1212	1550	1947	1578.89	306.84	80	1104	1580	1873	1531.62	322.79
33	54	1258	1680	1887	1633.7	303.12	84	1383	1750	2278	1820.82	380.85
34	70	1489	1922.5	2302	1944.5	332.03	136	1520	1885	2255	1891.92	306.12
35	90	1550	1950	2301	1933.89	301.72	178	1667	2025	2560	2060.86	344.94
36	134	1550	2250	2585	2143.02	416.99	236	1645	2200	2570	2164.37	391.02
37	136	1775	2240	2790	2248.53	430.99	322	1840	2350	2800	2340.81	384.96
38	146	1895	2400	2895	2419.79	397.78	276	1985	2450	3040	2469.31	409.96
39	82	1830	2350	3000	2349.88	419.67	192	1825	2500	3094	2485.16	470.86
40	42	1924	2300	2886	2364.52	423.32	44	2109	2565	3024	2576.82	407.95
41	16	1395	1960	2650	2013.75	553.98	16	1925	2470	2895	2410	429.33
							6	1960	2850	3030	2613.33	524.96

*P10* 10th centile, *P50* 50th centile, *P90* 90th centile

**Table 2:** Smoothed birthweight (g) centiles by gestational age (weeks) for twins according to chorionic placentation

		Monochorionic								Dichorionic						
GA	N	P5	P10	P25	P50	P75	P90	P95	N	P5	P10	P25	P50	P75	P90	P95
24	4	435	478	550	627	702	768	807	2	337	391	461	524	587	657	713
25	8	503	553	636	725	812	889	934	2	426	490	574	652	729	813	877
26	8	570	628	721	823	921	1008	1059	4	516	589	687	779	870	967	1040
27	2	637	701	806	919	1029	1126	1183	12	610	692	804	910	1015	1125	1205
28	6	707	778	894	1019	1141	1249	1312	12	709	799	924	1045	1165	1288	1376
29	26	782	861	990	1129	1264	1383	1453	16	810	908	1047	1184	1319	1455	1551
30	18	873	961	1104	1259	1409	1542	1621	26	914	1020	1173	1324	1475	1624	1728
31	18	974	1073	1233	1405	1574	1722	1810	50	1020	1134	1301	1468	1633	1796	1907
32	54	1081	1190	1367	1559	1746	1911	2008	80	1128	1250	1431	1613	1794	1971	2089
33	54	1187	1307	1502	1712	1917	2098	2205	84	1238	1368	1563	1761	1957	2148	2274
34	70	1293	1424	1636	1865	2089	2286	2402	136	1347	1485	1693	1907	2119	2323	2457
35	90	1393	1533	1762	2009	2250	2462	2587	178	1454	1600	1821	2050	2277	2494	2635
36	134	1489	1639	1883	2147	2404	2631	2765	236	1557	1709	1943	2187	2428	2657	2805
37	136	1569	1727	1985	2263	2535	2774	2915	322	1653	1812	2057	2314	2569	2809	2963
38	146	1624	1788	2055	2343	2624	2872	3018	276	1739	1904	2160	2428	2695	2946	3105
39	82	1647	1815	2087	2381	2666	2919	3069	192	1817	1987	2251	2531	2808	3068	3232
40	42	1646	1819	2096	2391	2680	2937	3090	44	1890	2064	2337	2626	2913	3181	3349
41	16	1613	1801	2091	2391	2684	2952	3119	16	1960	2138	2419	2718	3014	3290	3462
42	0								6	2028	2210	2499	2807	3113	3396	3573

*P5* 5th centile, *P10* 10th centile, *P25* 25th centile, *P50* 50th centile, *P75* 75th centile, *P90* 90th centile, *nd P95* 95th centile

## Discussion

In this study, we constructed new birth weight centile curves for twins born in South India. We have presented centile curves by chorionic placentation to facilitate consideration of chorionicity in the assessment of twin births.

The overall pattern of change in birthweight over gestational age was characterized by a rapid change in weight till 38 weeks and reduction in change then onwards. Given that it is increasingly possible to determine chorionicity prenatally, it is important to consider placental chorionicity in the assessment of growth in twins. Our comparison of centile curves by chorionicity showed that birth weights of monochorionic twins were lower than dichorionic twins in gestational ages between 28 and 42 weeks. This could be explained by the increased demands with advancing gestational age in monochorionic twins which share a common placenta and this heightened demand may not be met as adequately as in dichorionic twins- leading to the difference between two groups.

Previous studies on distribution of birth weights in India have mainly been based on singleton births. Birth weights from our study were consistently lower than those of singletons [8]; the differences were approximately 500 g between gestational ages 32 and 42 weeks. This difference was similar to that seen in other published studies on the birth weight centile curves for twins [4, 6].

The data presented here is based on the largest sample size reported till date from India. However, in developing settings like India, it is considerably difficult to obtain precise obstetrical records on measurements at birth for a large number of twins, as there are not many population based twin registries. The new birth weight curves may provide useful evidence for better understanding the birth weight of twins born in South India. For instance, it could serve as a useful tool for clinicians to evaluate and assess the birth weight of newborn twins. Additionally, this new centile curves should be a useful for epidemiologic research on twins related to determination of geographic differences, temporal trends and etiologic determinants of distribution of birth weight.

One of some limitations of this study is that the data were drawn from a tertiary care hospital, and hence it may restrict the generalizability of our results. However, given that twin pregnancies are considered as high-risk and often referred to tertiary care hospitals, the problems related to generalizability might be less likely. Another limitation is the measurement of gestational age using dates of last menstrual period, which suffers from recall bias. We believe our estimates are likely to be improved with the use of early ultrasound to correct estimates of gestational age. Also, data on birthweight and placental chorionicity captured during the course of routine clinical care may not be as precise as measurements under more controlled research settings. Thus, for example, we were not able to ascertain the extent of intra or inter observer variability. Additionally, the number of infants in extreme gestational ages was not sufficiently larger to enable accurate estimation of centiles. Further, in our study, the inclusion criteria resulted in a more general reference for birth weight, describing the variation in birth weight within a reference population and did not delineate variation that can be considered ‘ideal’ or of ‘desirable targets’. Despite these limitations, our study will add to the existing scanty literature on birth weight distributions for twin births and will provide basis for future epidemiological studies on twins from this region.

## Conclusion

The use of population specific birth weight centile curves will better aid both the clinician and researcher in the assessment of the birth weight of twins. Further, we recommend that assessment in twins consider placental chorionicity. The charts will provide a benchmark to examine the birth weight of twins in relation to other twins born of same gestational age, and would serve as a baseline for future epidemiological research studies. Future work will be to assess whether the infants identified in this way are those with high risk for poor perinatal outcomes, such as stillbirth and neonatal death.

## Abbreviation

GAMLSS: generalized additive models for location scale and shape
